# An evaluation of statistical differential analysis methods in single-cell RNA-seq data

**DOI:** 10.21203/rs.3.rs-2670717/v1

**Published:** 2023-03-23

**Authors:** Dongmei Li, Martin Zand, Timothy Dye, Maciej Goniewicz, Irfan Rahman, Zidian Xie

**Affiliations:** 1 Clinical and Translational Science Institute, School of Medicine and Dentistry, University of Rochester, 265 Crittenden Boulevard CU 420708, 14642 Rochester, NY, US.; 2 Department of Medicine, University of Rochester Medical Center, 601 Elmwood Ave, Box 675, 14642 Rochester, NY, US.; 3 Department of Obstetrics and Gynecology, University of Rochester, 500 Red Creek Drive Suite 220, 14623 Rochester, NY, US.; 4 Department of Health Behavior, Roswell Park Comprehensive Cancer Center, 14203 Buffalo, NY, US.; 5 Department of Environmental Medicine, University of Rochester, 14623 Rochester, NY, US.

**Keywords:** Single-cell RNA-seq, Differential analysis, AUROC curve

## Abstract

**Background:**

Single-cell RNA Sequencing is gaining popularity in recent years. Compared to bulk RNA-Seq, single-cell RNA Sequencing allows the gene expression being measured within individual cells instead of mean gene expression levels across all cells in the sample. Thus, cell-to-cell variation of gene expressions could be examined. Gene differential expression analysis remains the major purpose in most single-cell RNA sequencing experiments and many methods have been developed in recent years to conduct gene differential expression analysis for single-cell RNA sequencing data.

**Results:**

Through simulation studies and real data examples, we evaluated the performance of five open-source popular methods used for gene differential expression analysis in single-cell RNA sequencing data. The five methods included DEsingle (Zero-inflated negative binomial model), Linnorm (Empirical Bayes method on transformed count data using the limma package), monocle (An approximate Chi-Square likelihood ratio test), MAST (A generalized linear hurdle model), and DESeq2 (A generalized linear model with empirical Bayes approach and also commonly used for bulk RNA sequencing differential express analyses). We assessed the false discovery rate (FDR) control, sensitivity, specificity, accuracy, and area under the receiver operating characteristics (AUROC) curve for all five methods under different sample sizes, distribution assumptions, and proportions of zeros in the data.

**Conclusions:**

We found the MAST method performed the best among the five methods compared with the largest AUROC values across all tested sample sizes and different proportion of truly differential expressed genes, when the data followed negative binomial distributions. When the sample size increased to 100 in each group, the MAST method showed the best performance with the highest AUROC regardless of the data distributions. If the excess zeros were first filtered out before the gene differential analyses, the DESingle, Linnorm, and DESeq2 performed relatively better than the MAST and the monocle methods with higher AUROC values.

## Background

Single-cell RNA sequencing becomes increasingly popular in biological and biomedical research to examine gene regulatory relationships, identify cell populations and senescent cells in the aging process, and gene expression differences associated with different treatments or diseases ([1, 2]). Different from bulk RNA sequencing experiments, single-cell RNA sequencing experiments isolate and sequence individual cells extracted from normal tissues or tissues with diseases ([3–7]). Single-cell RNA sequencing will sequence genes in each cell and compare gene expressions between cells with different treatments or different types of cells ([8–10]). Based on gene expression levels, individual cells could be clustered into different populations, and cells within the same population might share some common characteristics ([7]). Compare to bulk RNA-seq which measures the average level of gene expression of multiple cells, single-cell RNA-seq allows us to understand gene expression patterns within the cell ([7–9]). Single-cell RNA-seq could also help us identify cell heterogeneity, cell population, and sub-population, identify specific cells such as senescence cells, as well as examine the effects of low copy mRNA distributions and transcription regulation ([7–9]).

Differential analyses are one of the main gene-level analyses in single-cell RNA-seq data. Although the commonly used differential analysis methods for bulk RNA sequencing differential analysis, such as DESeq2 ([11]) and edgeR ([12]), are still used for single-cell RNA sequencing differential analysis, more methods have been proposed to specifically analyze the single-cell RNA sequencing data, considering the large proportion of zeros and multimodal properties of the single-cell RNA sequencing data. Examples of proposed single-cell RNA-seq differential analyses methods include SCDE ([13]), MAST ([14]), scDD ([15]), BPSC ([16]), DEsingle ([17]), SigEMD ([18]), SC2P ([19]), CRE ([20]), DECENT ([21]), D3E ([22]), EMDomics ([23]), monocle ([24, 25]), Linnorm ([26]), and Discriminative Learning ([27]). A previous simulation study compared six single-cell RNA-seq differential analysis methods (MAST ([14]), SCDE ([13]), monocle ([24]), D3E ([22]), DESeq ([28]), edgeR ([12])) regarding their precision, recall rate, Area under the Recall Precision Curve (AUPRC), and the mean number of differential expressed genes detected ([5]). Results from both simulated (generated from negative binomial distribution) and real data in the above comparisons found a large difference in the performance of the methods compared and no single method is uniformly better than other methods ([5]). A recent simulation study compared the performance of six single-cell RNA sequencing differential analysis methods (MAST ([14]), DESingle ([17]), sigEMD ([18]), scDD ([15]), BPSC ([16]), and CRE ([20])) in terms of their true positive rate (TPR), false positive rate (FPR), observed false discovery rate (FDR), area under the receiver operating characteristic curve (AUROC), and the total number of differential expressed genes ([29]). The simulation studies simulated data based on gamma-Poisson distribution and set the proportion of truly differential expressed genes at 5% for mean shifts between groups ([29]). The results of the simulation studies did not find a uniformly better method among the six methods compared ([29]). A comparison of the reproducibility of multiple single-cell RNA sequencing differential analysis methods (BPSC, DEsingle ([17]), MAST ([14]), monocle ([24]), DESeq2 ([11]), edgeR ([12]), limmatrend ([30]), t-test, Wilcoxon signed rank test) found edgeR and monocle have less reproducibility than other differential analysis methods compared and all other methods have similar reproducibility ([31]).

In this investigation, we aim to evaluate the statistical differential analysis methods commonly used in single-cell RNA sequencing data analyses such as DESeq2, MAST, monocle, Linnorm, and DEsingle packages in the statistical analysis software R/Bioconductor. The performance of those statistical differential analysis methods will be indicated by false discovery rate (FDR) control, sensitivity, specificity, accuracy, and AUROC resulting from analyzing the same data set through simulation studies and real data examples. Different from previous simulation studies that compare the performance of single-cell RNA sequencing differential analysis methods, our simulation studies considered three different data generation distributions including negative-binomial, Poisson, and lognormal distributions with various proportions of truly differentially expressed genes and sample sizes in each group. Our simulation studies also considered two situations with and without excess zeros in the single-cell RNA-seq data to consider a possible practice of removing excess zeros before the differential analyses. We expect our evaluation could provide some guidance for investigators to choose the appropriate method for their single-cell RNA sequencing data analyses in different situations.

## Methods

### Performance Indicators

Multiple testing is an important issue in multiple hypotheses testing, especially in genomics and genetics data analyses, as the probability of at least one false rejection will be over 99% as the number of hypotheses increases to 90 or beyond. In genomics and genetics data analyses, the number of null hypotheses is usually over millions or tens of millions. Thus, controlling multiple testing error rates is very important in single-cell RNA sequencing analyses. Table 1 lists the possible outcomes when we test m null hypotheses simultaneously with m denoting the total number of genes in single-cell RNA sequencing data. Among the m genes, $m_{0}$ denotes the number of non-differential expressed genes and $m_{1}$ denotes the number of differentially expressed genes. Out of m genes, R denotes the number of total rejections. Out of $m_{0}$ genes, V denotes the number of false rejections and U denotes the number of true rejections. Out of $m_{1}$ genes, T denotes the number of differentially expressed genes that are not rejected and S denotes the number of differentially expressed genes that are rejected. The false discovery rate (FDR), sensitivity, specificity, accuracy, true positive rate (TPR), and false positive rate (FPR) are defined based on the following formulas.

$$FDR=E(\frac{V}{R})Pr(R>0)$$

$$Sensitivity=E(\frac{S}{m_{1}})$$

$$Specificity=E(\frac{U}{m_{0}})$$

$$Accuracy=E(\frac{U+S}{m})$$

$$TPR=\frac{S}{S+T}$$

$$FPR=\frac{V}{V+U}$$

A receiver operating characteristic (ROC) curve is a plot of TPR vs. FPR at different classification thresholds and AUC is a measurement of the entire two-dimension area under the ROC curve. AUC under the ROC curve (AUROC) denotes an aggregate performance and could be calculated using the AUC function in the pROC package in R.

### DEsingle

DEsingle uses the Zero-Inflated Negative Binomial (ZINB) model to describe the read counts and excess zeros in single-cell RNA sequencing data ([17]). The count data for *g*th gene in a group of cells are assumed to follow ZINB distribution:

$$Pr\left(Y_{g}=y|\theta ,r,p\right)=\theta \times I\left(y=0\right)+\left(1-\theta \right)\times f_{NB}\left(r,p\right),$$

where θ is the proportion of constant zeros of gene g in the group of cells, I(y=0) is an indicator function, $f_{NB}(r,p)$ is the probability mass function of Negative Binomial distribution with parameters r and p. DEsingle uses likelihood-ratio tests for gene differential analysis.

### Linnorm

Linnorm proposed a novel normalization and transformation method for single-cell RNA-seq analysis ([26]). The normalization and transformation parameters are calculated based on stably expressed genes across different cells. The moderated *t* test statistics in the limma package are used for differential analysis through the empirical Bayes approach.

### monocle

The monocle method published by Qiu et. al. in 2017 uses a census algorithm to convert relative RNA-seq expression levels into relative transcript counts without the need for experimental spike-in controls ([25]). The census algorithm calculates the total number of single-mRNA genes and divides this number by the fraction of the library contributed by them to estimate the total number of captured mRNAs in the cell and then rescales the transcript per million (TPM) in single-cell values into mRNA counts for each gene. monocle tests gene differential analysis using a likelihood ratio test by comparing a generalized linear model with additional effect to a reduced generalized linear model based on negative binomial distributions.

### MAST

MAST proposes a hurdle model approach for scRNA-seq data analysis ([14]). In the scRNA-seq expression data $Y_{ig}$, the rate of expression and the level of expression for the expressed cells are assumed conditionally independent for each gene g([14]). MAST use an indicator variable $Z_{ig}$ to denote whether gene g is expressed in cell *i* ($z_{ig}=0$ if $y_{ig}=0$ and $z_{ig}=1$ if $y_{ig}>0$). MAST fits a logistic regression for the discrete variable Z and a normal distributed linear model for the continuous variable (Y|Z=1) independently.

$$logit(Pr(Z_{ig}=1)=X_{i}\beta_{g}^{D},\;Pr(Y_{ig}=y|Z_{ig}=1)=N(X_{i}\beta_{g}^{C},\sigma_{g}^{2})$$

### DESeq2

DESeq2 are commonly used differential analysis methods for bulk RNA sequencing differential analysis that uses a generalized linear model approach to accommodate complex study designs ([11]). DESeq2 uses a logarithm link between relative gene abundance and the design matrix. DESeq2 integrates the dispersion estimate and fold change estimate using the empirical Bayes approach and tests the differential expression using a Wald test.

### Simulation Setup

RNA sequencing count data are generated from negative binomial (NB), Poisson, and lognormal distributions using the RnaXSim function in R. The means and variance are generated using two sets of real RNA sequencing count data with estimated different proportions of zeros in the data. 1000 genes and 20 independent simulations. Fraction of differentially expressed genes (*π*_1_) were set at 5%, 10%, 20%, 30%, 40% and 50%. Sample sizes are 5, 10, and 15 in each group with two-group comparisons.

We considered two simulation situations: one with excessive zeros in the single-cell RNA sequencing data set and one without excessive zeros in the single-cell RNA sequencing data set. Our first simulation study focused on single-cell RNA sequencing data with excessive zeros. The proportions of zeros in the simulated data set ranged from 4% to 6% for the negative binomial distribution, 2% to 3% for the Poisson distribution, and 1% for the lognormal distribution. The proportions of all zeros are pretty constant across different sample sizes. In our second simulation study, the proportions of all zeros in the simulated data set are less than 0.4% for the negative binomial distribution and close to zero for the Poisson and the lognormal distribution. The proportions of all zeros are also pretty constant across different sample sizes in our second simulation study.

## Results

### Simulation Study One

**Figure 1:**
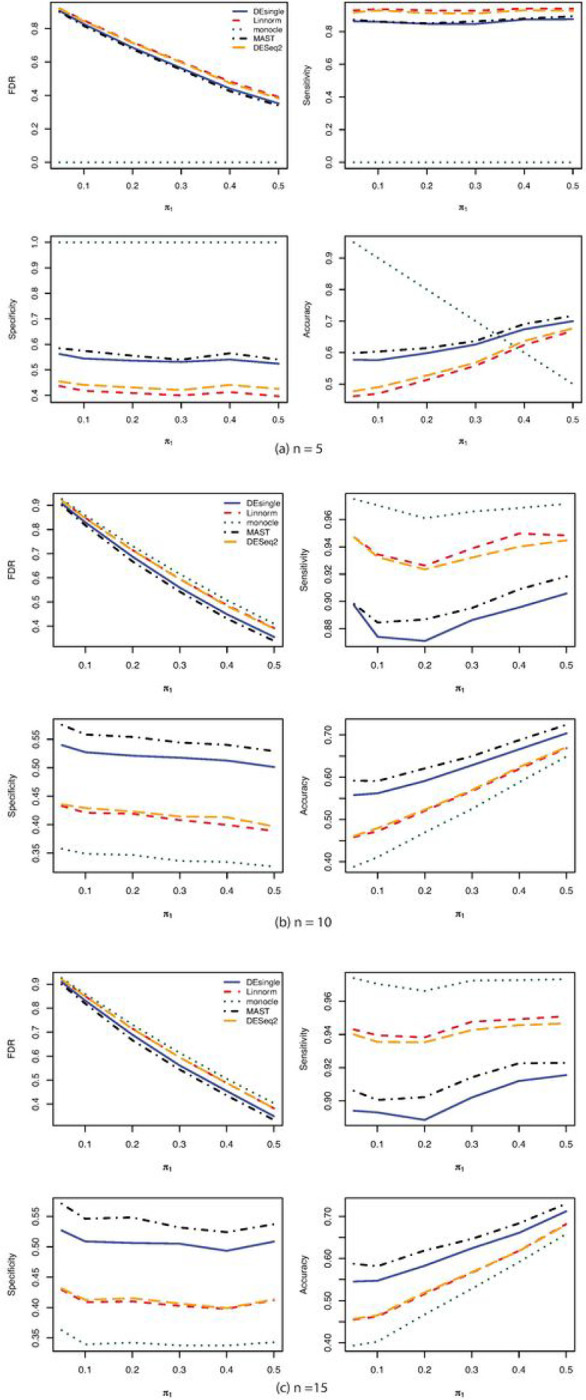
FDR, Sensitivity, Specificity, and Accuracy comparisons for sample size 5, 10, and 15 in each group with negative binomial (NB) distribution. (a) FDR, Sensitivity, Specificity, and Accuracy comparisons for sample size 5 in each group with NB distribution and relatively large proportion of zeros in the data; (b) FDR, Sensitivity, Specificity, and Accuracy comparisons for sample size 10 in each group with NB distribution and relatively large proportion of zeros in the data; (c) FDR, Sensitivity, Specificity, and Accuracy comparisons for sample size 15 in each group with NB distribution and relatively large proportion of zeros in the data.

**Figure 2:**
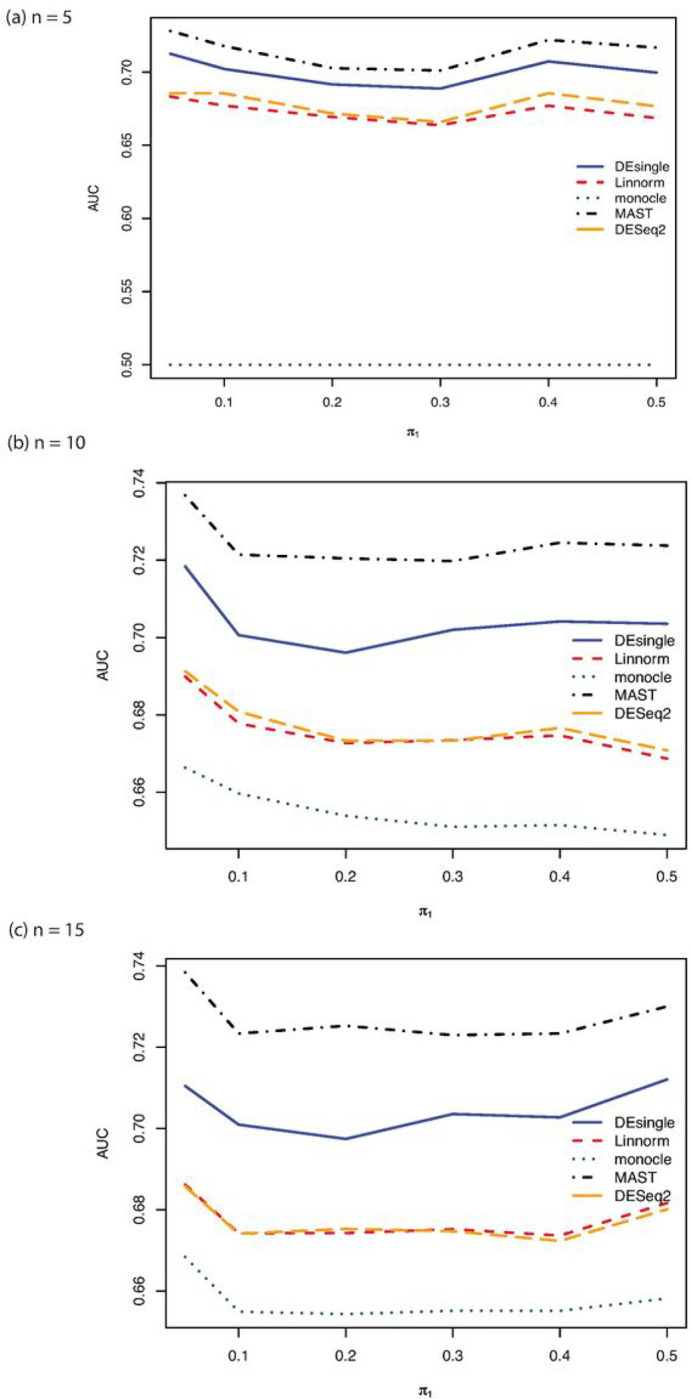
AUROC comparisons for sample size 5, 10, and 15 in each group with NB distribution. (a) AUROC comparisons for sample size 5 in each group with NB distribution and relatively large proportion of zeros in the data; (b) AUROC comparisons for sample size 10 in each group with NB distribution and relatively large proportion of zeros in the data; (c) AAUROC comparisons for sample size 15 in each group with NB distribution and relatively large proportion of zeros in the data.

When the single-cell RNA sequencing count data were generated from the negative binomial distribution, the FDR control, sensitivity, specificity, and accuracy were compared among the five commonly used methods for sample sizes of 5, 10, and 15 in each group (Figure 1). The MAST and the DEsingle methods have relatively better FDR control, specificity, and accuracy than the DESeq2 and the Linnorm methods across all sample sizes. The sensitivities of the Linnorm and DESeq2 methods were slightly better than the MAST and DESingle methods. The monocle method behaved very differently from other methods. The monocle method was much more sensitive to sample size than other compared methods. The sensitivity of the monocle method was almost zero when the sample size was only 5 in each group and increased to over 96% when the sample size increased to 10 in each group. However, the specificity and accuracy of the monocle method were the lowest among all the five methods. As the proportions of truly deferentially expressed genes increased, both the FDR control and the accuracy got better. The sensitivity and specificity were relatively constant as the proportions of truly differentially expressed genes increased. The AUROC ranked the performance of the five methods compared with the MAST method as the best one followed by the DEsingle, DESeq2, Linnorm, and monocle (Figure 2).

**Figure 3:**
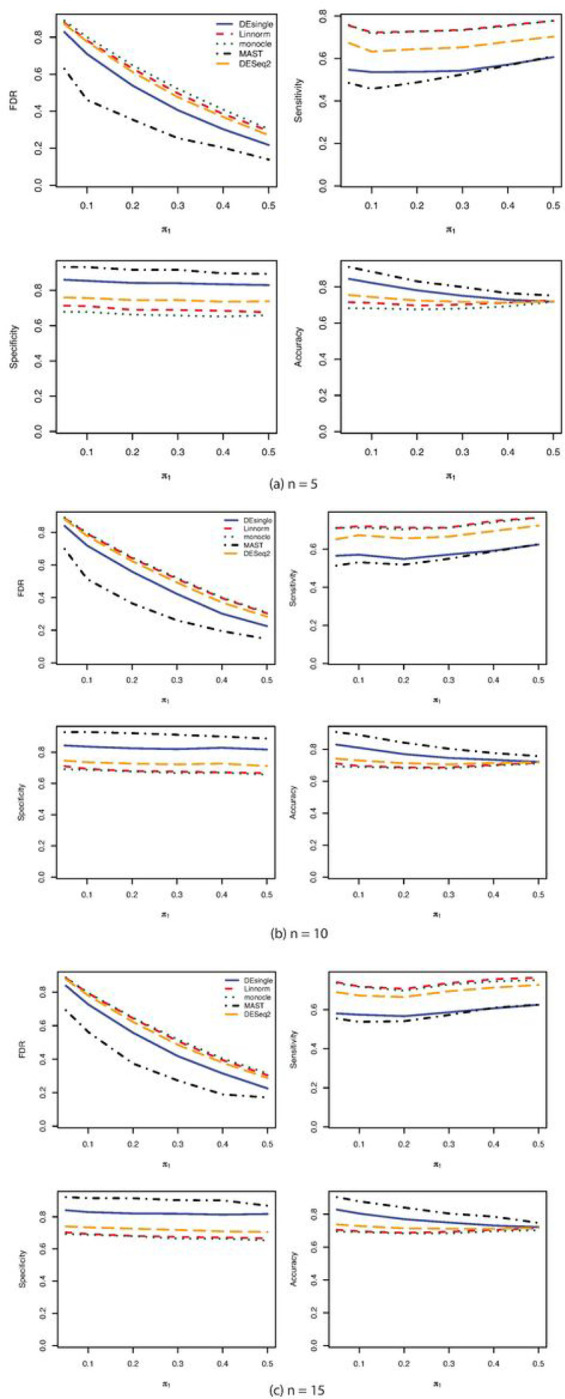
FDR, Sensitivity, Specificity, and Accuracy comparisons for sample size 5, 10, and 15 in each group with Poisson distribution. (a) FDR, Sensitivity, Specificity, and Accuracy comparisons for sample size 5 in each group with Poisson distribution and relatively large proportion of zeros in the data; (b)FDR, Sensitivity, Specificity, and Accuracy comparisons for sample size 10 in each group with Poisson distribution and relatively large proportion of zeros in the data; (c) FDR, Sensitivity, Specificity, and Accuracy comparisons for sample size 15 in each group with Poisson distribution and relatively large proportion of zeros in the data.

**Figure 4:**
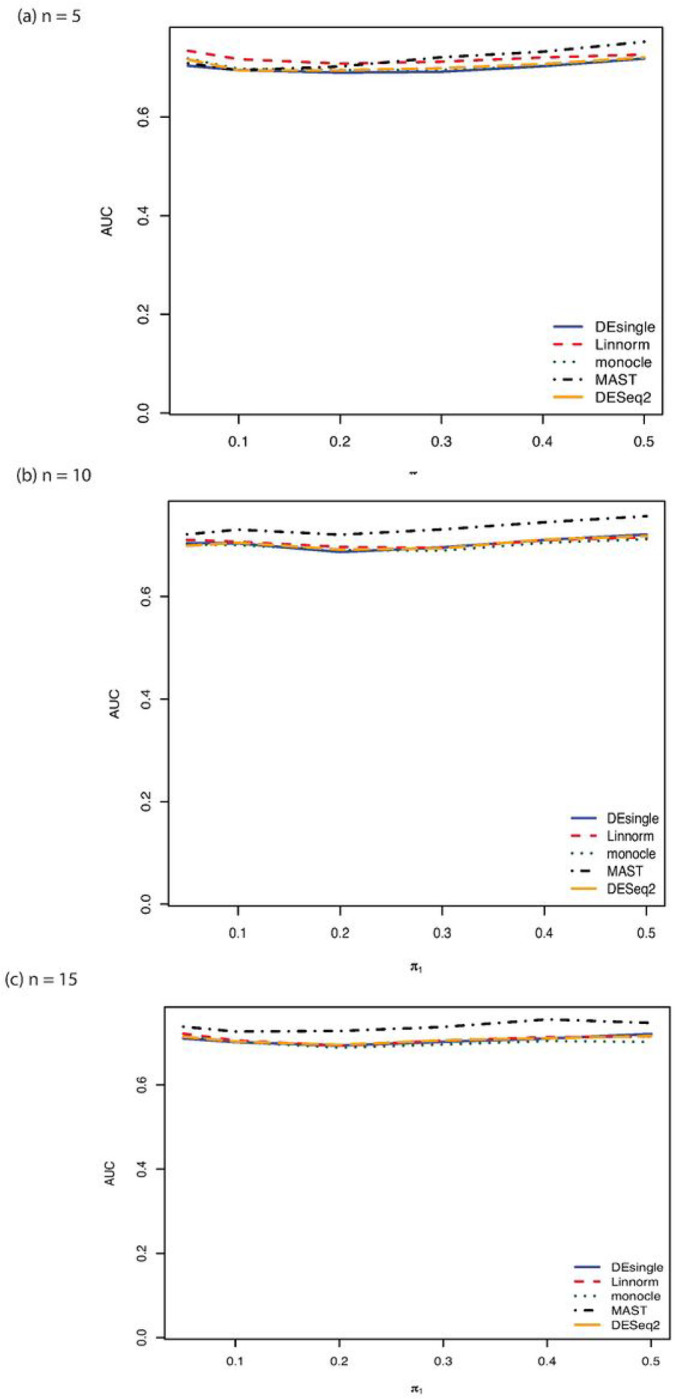
AUROC comparisons for sample size 5, 10, and 15 in each group with Poisson distribution. (a) AUROC comparisons for sample size 5 in each group with Poisson distribution and relatively large proportion of zeros in the data; (b) AUROC comparisons for sample size 10 in each group with Poisson distribution and relatively large proportion of zeros in the data; (c) AUROC comparisons for sample size 15 in each group with Poisson distribution and relatively large proportion of zeros in the data.

When the distribution assumption of the single-cell RNA sequencing data changed from negative binomial distribution to Poisson distribution, the FDR control, sensitivity, specificity, and accuracy for samples sizes of 5, 10, and 15 in each group were shown in Figure 3. When the sample size was as small as 5 in each group, the MAST method showed the best FDR control, the highest specificity, and the highest accuracy, followed by the DEsingle, the DEseq2, the Linnorm, and the monocle method. The monocle method and the Linnorm method had almost equal sensitivity and both their sensitivities were higher than the DESeq2, the DEsingle, and the MAST methods. We observed the same trends in the FDR, sensitivity, specificity, and accuracy as the sample size increased to 10 and 15 in each group. The performance of the monocle method was not affected by the small sample size when the data were generated from Poisson distributions. Only FDR control seemed to get better as the proportions of truly differential expressed genes increased. The other performance indicators seemed relatively invariant to the proportions of truly differential expressed genes. When the sample size was as small as 5 in each group, the Linnorm method had relatively higher AUC under the ROC curve than all other methods with the proportions of deferentially expressed genes lower than 20%. As the sample size further increased to 10 and 15 in each group, the MAST method showed the largest AUC under the ROC curve among all the methods compared (Figure 4).

**Figure 5:**
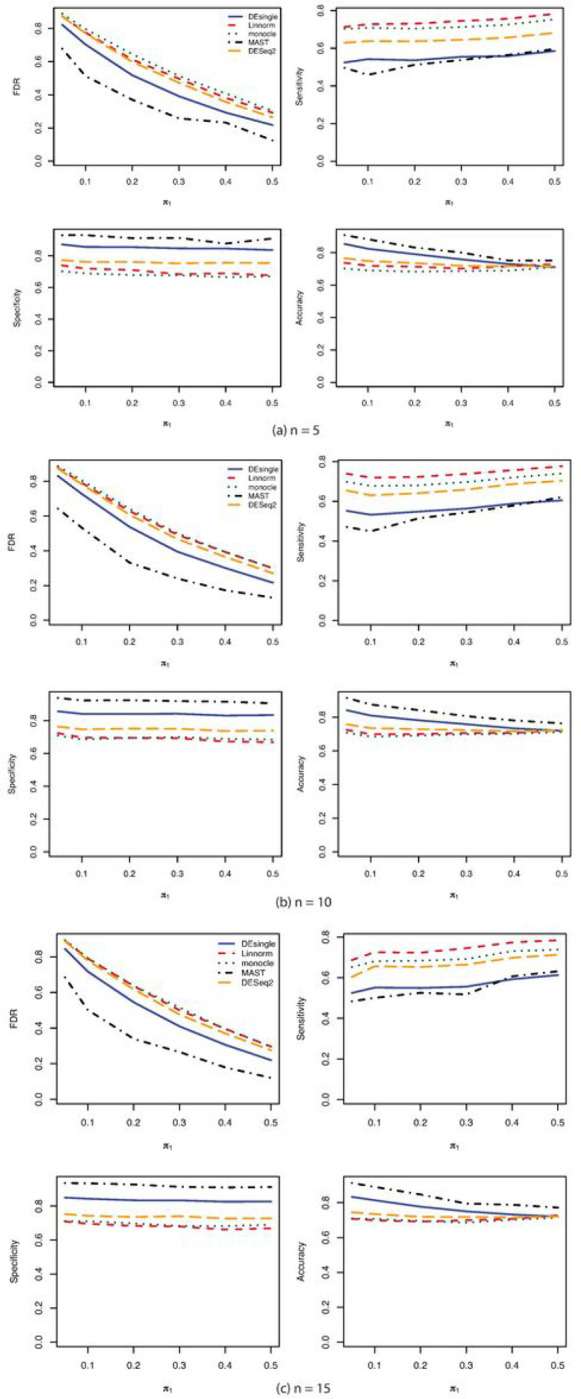
FDR, Sensitivity, Specificity, and Accuracy comparisons for sample size 5, 10, and 15 in each group with log normal distribution. (a) FDR, Sensitivity, Specificity, and Accuracy comparisons for sample size 5 in each group with log normal distribution and relatively large proportion of zeros in the data; (b) FDR, Sensitivity, Specificity, and Accuracy comparisons for sample size 10 in each group with log normal distribution and relatively large proportion of zeros in the data; (c) FDR, Sensitivity, Specificity, and Accuracy comparisons for sample size 15 in each group with log normal distribution and relatively large proportion of zeros in the data.

**Figure 6:**
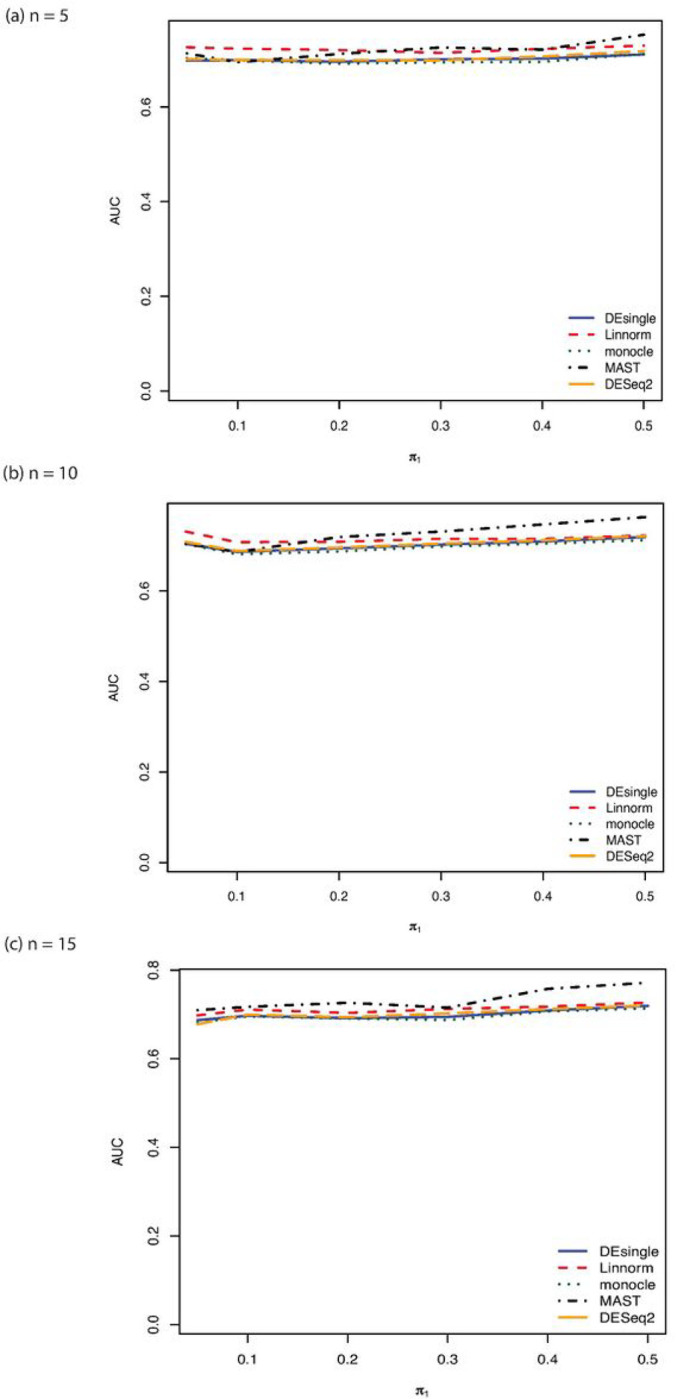
AUROC comparisons for sample size 5, 10, and 15 in each group with log normal distribution. (a) AUROC comparisons for sample size 5 in each group with log normal distribution and relatively large proportion of zeros in the data; (b) AUROC comparisons comparisons for sample size 10 in each group with log normal distribution and relatively large proportion of zeros in the data; (c) AUROC comparisons for sample size 15 in each group with log normal distribution and relatively large proportion of zeros in the data.

After the single-cell RNA sequencing data distribution assumption changed to lognormal distribution, we observed similar trends as that for the Poisson distribution in terms of the FDR control, sensitivity, specificity, and accuracy (Figure 5). The MAST method showed the best FDR control, specificity, and accuracy followed by the DEsingle, DESeq2, Linnorm, and monocle method when the sample size was as small as 5 in each group. The Linnorm method showed the highest sensitivity among all the methods compared, followed by the monocle, DESeq2, DEsingle, and MAST. When the sample size further increased to 10 and 15 in each group, we observed the same trend except that the differences between the Linnorm and the monocle methods in FDR control, specificity, and accuracy becomes smaller than that when the sample size was 5 in each group. For the AUC under the ROC curve, we noticed that the Linnorm method had the largest AUC values than other methods compared when the sample size was as small as 5 in each group and the proportion of differential expressed genes was lower than 20% (Figure 6). The MAST method had the largest AUC values when the portion of differentially expressed genes was larger than 20%. When the sample size increased to 10 in each group, the Linnorm method had the largest AUC values when the proportion of differentially expressed genes was lower than 15%. The MAST method had the largest AUC values when the proportion of differential expressed genes was greater than 15%. Interestingly, when the sample size further increased to 15 in each group, the MAST method had the largest AUC under the ROC curve for the entire range of the proportions of differentially expressed genes.

**Figure 7:**
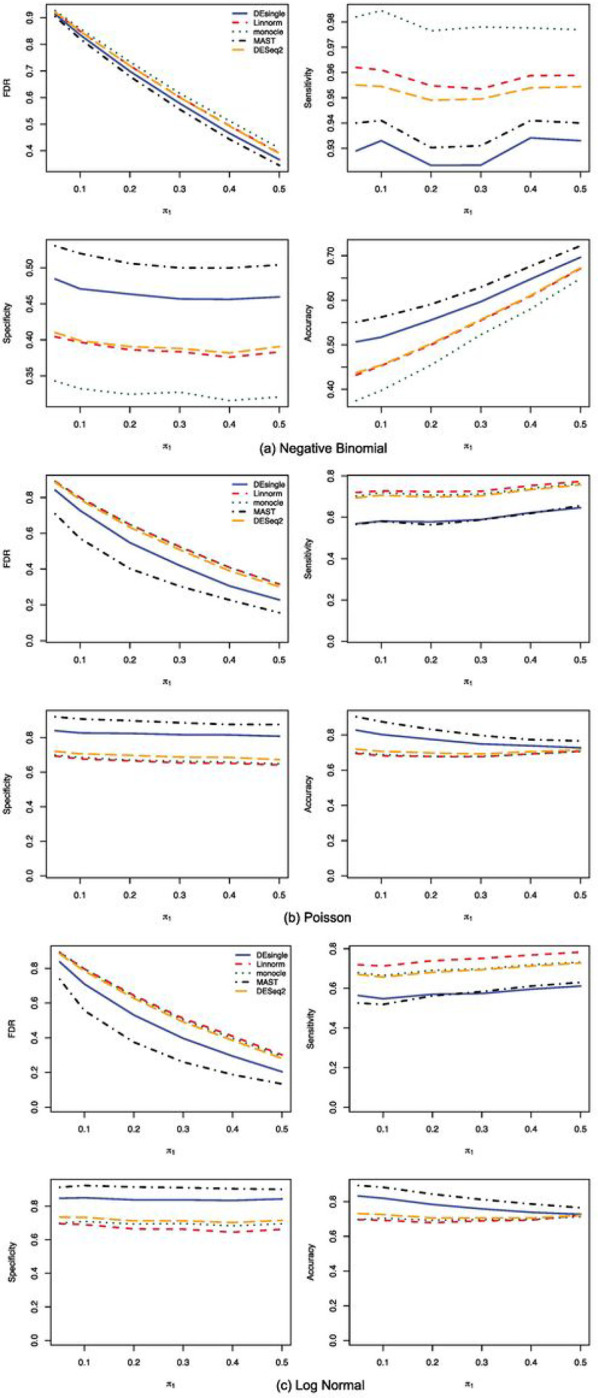
FDR, Sensitivity, Specificity, and Accuracy comparisons for sample size 100 in each group with NB, Poisson, and log normal distributions. (a) FDR, Sensitivity, Specificity, and Accuracy comparisons for sample size 100 in each group with NB distribution and relatively large proportion of zeros in the data; (b) FDR, Sensitivity, Specificity, and Accuracy comparisons for sample size 100 in each group with Poisson distribution and relatively large proportion of zeros in the data; (c) FDR, Sensitivity, Specificity, and Accuracy comparisons for sample size 100 in each group with log normal distribution and relatively large proportion of zeros in the data.

**Figure 8:**
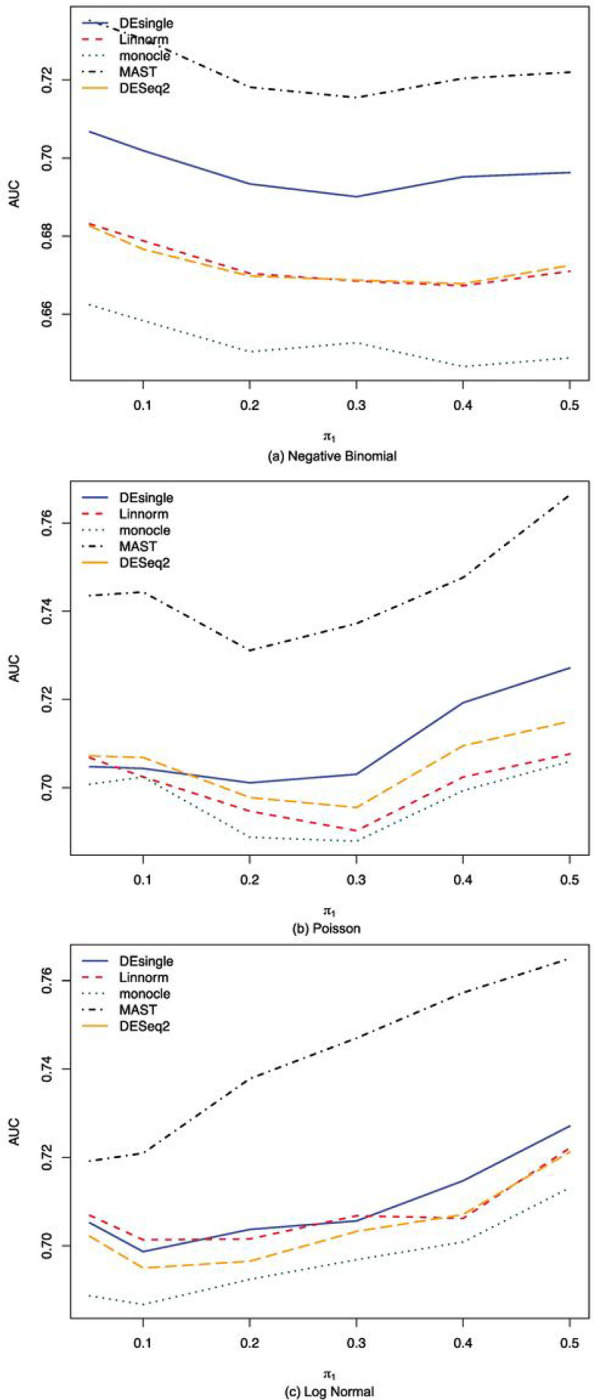
AUC under ROC curves comparisons for sample size 100 in each group with NB, Poisson, and log normal distribution. (a) AUROC comparisons for sample size 100 in each group with NB distribution and relatively large proportion of zeros in the data; (b) AUROC comparisons comparisons for sample size 100 in each group with Piosson distribution and relatively large proportion of zeros in the data; (c) AUROC comparisons comparisons for sample size 100 in each group with log normal distribution and relatively large proportion of zeros in the data

When the sample size further increased to 100 in each group, we noticed that the MAST method showed the best FDR control, the highest specificity, and the highest accuracy among all the five methods compared, regardless of the data distribution assumptions (Figure 7). The monocle method had the highest sensitivity when the data followed negative binomial distributions, while its FDR control, specificity, and accuracy were the worst among the five methods compared (Figure 7a). When the data follows either Poisson or lognormal distributions, the performance of the monocle, the Linnorm, and the DESeq2 methods were close to each other (Figure 7b and Figure 7c). We also noticed that for all five methods, their sensitivities were the highest when the data follows a negative binomial distribution, while their specificity was higher for Poisson or lognormal distributed data than for negative binomial distributed data. The AUROC comparisons showed the MAST method had the best performance overall among all five methods compared, regardless of the data distributions (Figure 8). For data following negative binomial distributions, the DEsingle method was the second-best method considering the overall performance. The performances of the DESeq2 method and the Linnorm method were pretty close and the performance of the monocle method was the worst (Figure 8a). When the data followed either Poisson or lognormal distributions, the performance of the DEsingle, Linnorm, DESeq2, and monocle were very close with monocle showed slightly lower AUROC values than the other three methods (Figure 8b and Figure 8c).

### Simulation Study Two

Our second simulation study focused on the performance comparisons among the five commonly used single-cell RNA sequencing differential analysis methods when the proportion of zero counts was very small. For example, when the genes with many zero counts were filtered before the differential analyses. The Figure S1 showed the performance comparisons when the data were generated according to the negative binomial distribution. The MAST method showed the best FDR control and the highest specificity regardless of the proportions of differentially expressed genes. The accuracy of the MAST method was the highest when the proportion of differentially expressed genes was less than 20% and the DEsingle method had the highest accuracy when the proportion of differential expressed genes was larger than 20%. However, the sensitivity of the MAST method was much lower than other methods compared when the sample size was as small as 5 in each group. The monocle method had the worst FDR control, and the lowest specificity and accuracy among the five compared methods although the sensitivity of the monocle method was the highest. When the sample size increased to 10 in each group, the MAST method still had the best FDR control and the largest specificity followed by the DEsingle, Linnorm, DESeq2, and monocle. The sensitivity of the MAST method also increased compared to that when the sample size was 5 in each group. The accuracy of the MAST method was still the highest among the five methods when the proportion of differentially expressed genes was less than 30%, and just slightly less than the DEsingle method when the proportion of differentially expressed genes was over 30%. When the sample size further increased to 15 in each group, the MAST method still had the best FDR control and the highest specificity. The accuracy of the MAST method was also the highest among all the five methods compared across the entire range of proportions of differentially expressed genes. The sensitivity of the MAST method also improved to over 80%. The DEsingle method also showed very good performance following the MAST method. The AUC under the ROC curve showed that the overall performance of the DEsingle method was the best followed by the Linnorm, DESeq2, MAST, and monocle when the sample size was 5 in each group. When the sample size increased to 10 in each group, the MAST method had a better AUC value than the Linnorm, DESeq2, and monocle when the proportion of differential expressed gene was greater than 30%. When the sample size further increased to 15 in each group, the MAST method had better AUC than the Linnorm, DESeq2, and monocle methods across all proportions of differentially expressed genes. The AUC values of the DEsingle and the MAST methods were almost identical when the proportion of differential expressed genes was larger than 30% (Figure S2).

When the single-cell RNA sequencing data were generated from the Poisson distribution, the five methods compared behaved very differently (Figure S3). When the sample size was 5 in each group, the MAST method had the best FDR control and the highest specificity. The accuracy of the MAST method was relatively high when the proportion of differential expressed genes was less than 20%. However, the accuracy of the MAST method decreased as the proportion of differentially expressed genes increased. The MAST method had the lowest accuracy when the proportion of differential expressed genes was 50%. The sensitivity of the MAST method was almost zero across all proportions of differentially expressed genes. The DEsingle method had better FDR control than the DESeq2, Linnorm, and monocle methods, although the FDR level was over 30% when the proportion of differentially expressed genes was less than 10%. The DEsingle method had high specificity and the best accuracy among the five methods compared. However, the sensitivity of the DEsingle method was lower than 60%. The DESeq2 and the Linnorm methods had high FDR levels, especially when the proportion of differentially expressed genes was small. The overall sensitivity, specificity, and accuracy of the DESeq2 and the Linnorm methods were all over 70%. The monocle method had the worst FDR control, and the lowest specificity and accuracy when the proportion of differentially expressed genes was less than 40%. The sensitivity of the monocle method was over 70% and slightly less than the DESeq2 and Linnorm methods. When the sample size increased to 10 and 15 in each group, we observed a similar trend. The AUC under the ROC curve showed the overall performance of the five methods (Figure S4). The DESeq2 method had the best performance among the five methods compared, followed by the Linnorm, DEsingle, monocle, and MAST methods. The MAST method had the lowest AUC values among the five methods compared. The AUC values remained similar as the sample size increased to 10 and 15 in each group.

When lognormal distribution was used to generate the single-cell RNA sequencing data, the five methods behaved differently (Figure S5). When the sample size was 5 in each group, the MAST method had the best FDR control although the FDR controls from all methods were bad. The sensitivity of the MAST method was the lowest although the specificity of the MAST method was the highest. The accuracy of the MAST method was the highest when the proportion of differentially expressed genes was less than 20%. However, the accuracy of the MAST method decreased as the proportion of differentially expressed genes increased. The DEsingle, Linnorm, and DESeq2 methods had relatively good performance in terms of sensitivity, specificity, and accuracy. The monocle method had the worst FDR control, zero specificity, and the lowest accuracy, although its sensitivity was almost 100%. We observed a similar trend as the sample size increased to 10 and 15 in each group. The sensitivity of the MAST method improved as the sample size increased. When we examined the overall performance of the five methods using the AUC under the ROC curve, we observed that the DEsingle method performed the best, followed by the Linnorm, DESeq2, MAST, and monocle methods. The AUC values of the MAST method increased as the proportion of differentially expressed genes increased (Figure S6).

When the sample size increased to 100 in each group, we noticed that the MAST method still performed well when the data followed either negative binomial or lognormal distributions (Figure S7). However, the MAST method became the worst one when the data followed the Poisson distribution with almost zero sensitivity. The DEsingle method became the best method when the data followed a negative binomial distribution with slightly better performance than the MAST method, in terms of the FDR control, specificity, and accuracy. The monocle method had the worst performance among the five methods compared for either negative binomial distributed or lognormal distributed data with the worst FDR control and almost zero specificity. The AUROC comparisons showed that the DEsingle and the MAST methods had the largest AUROC value when the data followed negative binomial distribution (Figure S8). While the monocle method had the lowest AUROC values for negative binomial distributed data. When the data followed Poisson distribution, the DESeq2 method showed the best overall performance followed by the Linnorm, the DEsingle, the monocle, and the MAST methods. The MAST method had the lowest AUROC values across all proportions of truly differentially expressed genes. While for lognormal distributed data, the DEsingle method showed the best AUROC values followed by the Linnorm, the MAST, the DESeq2, and the monocle methods. The monocle method had the lowest AUROC values across different proportions of truly differential expressed genes for lognormal distributed data.

### Real Data Example

We downloaded the Islam single-cell RNA sequencing data with 92 samples from the GEO website with accession no. GSE29087 to examine the empirical power and the overlap of identified differentially expressed genes among the five differential analysis methods. The 92 samples of the Islam single-cell RNA sequencing data included 48 samples from mouse embryonic stem cells and 44 samples from mouse embryonic fibroblasts. All methods were used for selecting differentially expressed genes between the two types of cells from the total 14905 genes. The raw *p*-values from all methods were adjusted using the Benjamini-Hochberg procedure to control FDR at 5%.

**Figure 9:**
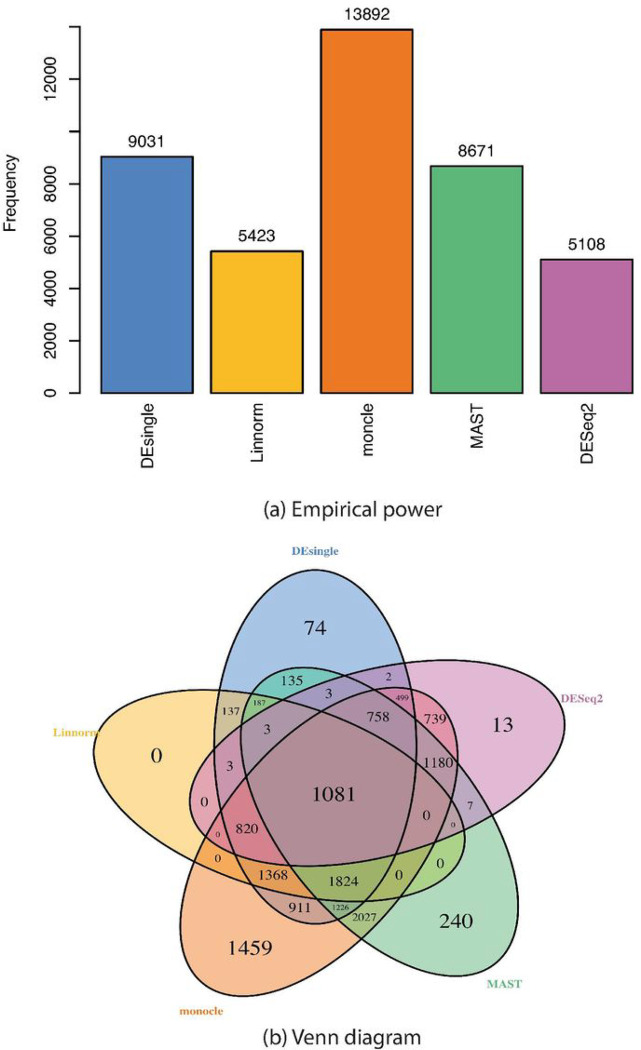
Empirical power and venn diagram of selected deferentially expressed genes from different single-cell RNA sequencing differential analyses methods. (a) Empirical power of different single-cell RNA sequencing differential analysis methods; (b) Venn diagram of selected deferentially expressed genes from different single-cell RNA sequencing differential analyses methods.

The empirical power comparison indicated that the monocle method had the largest empirical power followed by the DEsingle, MAST, Linnorm, and DESeq2 (Figure 9a). The Venn diagram of the identified differentially expressed genes from the five methods showed that only 1081 genes had been identified by all five methods (Figure 9b). The MAST method had the most number of differentially expressed genes (1824) that were identified by four methods, followed by the monocle (820), the DESeq2 (758), the DESeq2 (3), and the Linnorm (0) method. The monocle method had the largest number of differentially expressed genes (1459) that were not identified by the other four method, followed by the MAST (240), the DEsingle (74), DESeq2 (13), and the Linnorm (0) method.

## Discussions

In this investigation, we evaluated the performance of some commonly used single-cell RNA sequencing differential analysis methods such as DESeq2, MAST, monocle, Linnorm, and DEsingle, using both simulation studies and real data examples. In general, the MAST method performs relatively better than the other four methods used for gene differential analyses in single-cell RNA sequencing data, especially when the sample size is at least 100 in each group. The Linnorm method also performs well when the sample size is small such as 5, 10, or 15 in each group and the data follows a lognormal distribution. If one decides to first remove the excess zeros in the single-cell RNA sequencing data before the gene differential analyses, then the DESingle, Linnorm, and DESeq2 methods perform relatively well regardless of the distribution of the data.

In our simulation studies, we noticed that the FDR is not well controlled for all methods under all data distribution assumptions. The large estimated FDR values from all five differential analysis methods indicated the need for better differential analysis methods with satisfactory FDR control, especially with a small proportion of truly differentially expressed genes like 10% and a large sample size like 100 in each group. It seems that the FDR control improves with the increasing proportions of truly differential expressed genes, which might be because the total number of false rejections (*V* ) increased at a slower rate compared to the total number of rejections (*R*) in the FDR estimates. Both sensitivity and specificity seem relatively stable across different proportions of differential expressed genes, which might due to the similar increasing rate of the number of correctly identified positives (*S*) and correctly identified negatives (*U*) to the increasing rate of the number of true positives (*m*_1_) and true negatives (*m*_0_) as the proportion of truly differentially expressed genes increases. For all five methods compared, AUROC seems relatively stable across different proportions of differentially expressed genes. The relative stable AUROC values are expected as AUROC is a combined measure of TPR and FPR and there is a trade-off between TPR and FPR. The relatively larger AUROC values from the MAST method indicated its superior performance compared to the other four methods. The performance of different methods seems related to the proportion of total zeros in the data set. When the differential analysis methods were directly applied to normalized single-cell RNA sequencing data with excess zeros, methods modeling excess zeros showed better performance than methods that do not model excess zeros. While if the excess zeros were removed before the gene differential analyses, the commonly used bulk RNA sequencing differential analysis method, DESeq2, showed good performance. In addition, the DEsingle and Linnorm methods perform similarly to the DESeq2 method, which might due to the application of the empirical Bayes approach in all three methods. The performance of the monocle method seems to depend on data distributions and the proportion of total zeros in the data set. In most cases, the monocle method has the worst performance among the five methods compared, although the monocle method used in our simulation studies is already the improved version of the original monocle method.

In our real data examples, we observed that the monocle method had the largest empirical power with the greatest number of genes identified to be differentially expressed. In our simulation results, we showed that the monocle method had the largest sensitivity, worst FDR control, lowest specificity, and accuracy, when the sample size was 100 in each group and the data follows a negative binomial distribution. Thus, the largest empirical power could include both a large number of true positives and false positives, which was also indicated by the Venn diagram. From the Venn diagram, we noticed that the monocle method had the largest number of genes that were not identified by any of the other four methods, which indicated a higher probability of false positives than the other four methods. The poor FDR control from the monocle method is consistent with the results from a previous study, in which they used negative control real data and found the monocle method has the largest FDR among the single-cell gene differential analysis methods including monocle, MAST, DESeq2, DEsingle, edgeR, EMDomics, D3E, scDD, SCDE, SINCERA, and SigEMD ([32]). When the proportions of truly differentially expressed genes are small like in real data situations, the large FDR estimates from all the five evaluated methods in our simulation studies are another concern for the need for new methods to be developed with good FDR control. The large FDR estimates are also consistent with the investigation results in a recent study that evaluated the false discoveries in seven single-cell RNA sequencing differential analysis methods through simulation studies ([33]).

There are several limitations in our current investigation. First, our simulation studies only considered uni-modal situations and did not include bi-modal or multi-modal possibilities that might be the situations in real data examples. Second, our investigation only compared five commonly used gene differential analysis methods for single-cell RNA sequencing data. Although we showed that the MAST method seemed to be the best one among the five methods compared, we do not infer that the MAST method is the best gene differential analysis method for single-cell RNA sequencing data analyses as more methods have been developed for single-cell RNA sequencing differential analysis and further investigations are needed to evaluate the performance of those new methods.

## Conclusions

In conclusion, the hurdle model approach used in the MAST method seems to work well for gene differential analysis in single-cell RNA sequencing data with a certain proportion of excess zeros, regardless of the data distributions and the sample sizes. Among the five gene differential analysis methods (MAST, DEsingle, Linnorm, DESeq2, and monocle) we compared in this investigation, we recommend the MAST method over the other four methods for the downstream gene differential analysis in single-cell sequencing data that contain a large proportion of zero counts.

## Figures and Tables

**Table 1:** Possible outcomes from *m* hypotheses tests

	number not rejected	number rejected	

true null hypotheses	*U*	*V*	*m*_0_
non-true null hypotheses	*T*	*S*	*m*_1_
total	*m* – *R*	*R*	*m*

## Data Availability

The R code for simulations and real data example can be freely downloaded from Github website ”https://github.com/DongmeiLi2017/Single-cell-RNA-seq-Differential-Analyses-Comparisons”.
